# Stage‐specific regulation of Gremlin1 on the differentiation and expansion of human urinary induced pluripotent stem cells into endothelial progenitors

**DOI:** 10.1111/jcmm.15433

**Published:** 2020-05-28

**Authors:** Haixuan Chen, Zhen Zhang, Zhecun Wang, Quhuan Li, Hui Chen, Song Guo, Lin Bao, Zheng Wang, Wang Min, Qiuling Xiang

**Affiliations:** ^1^ Translational Medicine Center the First Affiliated Hospital Sun Yat‐sen University Guangzhou China; ^2^ Zhongshan School of Medicine Sun Yat‐sen University Guangzhou China; ^3^ Key Laboratory for Stem Cells and Tissue Engineering Center for Stem Cell Biology and Tissue Engineering Ministry of Education Sun Yat‐sen University Guangzhou China; ^4^ Department of Vascular Surgery the First Affiliated Hospital Sun Yat‐sen University Guangzhou China; ^5^ Institute of Biomechanics School of Bioscience and Bioengineering South China University of Technology Guangzhou China; ^6^ Department of Gynecology and Obstetrics Sun Yat‐sen Memorial Hospital Sun Yat‐sen University Guangzhou China; ^7^ Department of Genetics and Cell Biology Basic Medical College Qingdao University Qingdao China; ^8^ Interdepartmental Program in Vascular Biology and Therapeutics Department of Pathology Yale University School of Medicine New Haven CT USA

**Keywords:** differentiation, endothelial progenitors, expansion, Gremlin1, human urinary induced pluripotent stem cells

## Abstract

Human urinary induced pluripotent stem cells (hUiPSCs) produced from exfoliated renal epithelial cells present in urine may provide a non‐invasive source of endothelial progenitors for the treatment of ischaemic diseases. However, their differentiation efficiency is unsatisfactory and the underlying mechanism of differentiation is still unknown. Gremlin1 (GREM1) is an important gene involved in cell differentiation. Therefore, we tried to elucidate the roles of GREM1 during the differentiation and expansion of endothelial progenitors. HUiPSCs were induced into endothelial progenitors by three stages. After differentiation, GREM1 was obviously increased in hUiPSC‐induced endothelial progenitors (hUiPSC‐EPs). RNA interference (RNAi) was used to silence GREM1 expression in three stages, respectively. We demonstrated a stage‐specific effect of GREM1 in decreasing hUiPSC‐EP differentiation in the mesoderm induction stage (Stage 1), while increasing differentiation in the endothelial progenitors' induction stage (Stage 2) and expansion stage (Stage 3). Exogenous addition of GREM1 recombinant protein in the endothelial progenitors' expansion stage (Stage 3) promoted the expansion of hUiPSC‐EPs although the activation of VEGFR2/Akt or VEGFR2/p42/44MAPK pathway. Our study provided a new non‐invasive source for endothelial progenitors, demonstrated critical roles of GREM1 in hUiPSC‐EP and afforded a novel strategy to improve stem cell‐based therapy for the ischaemic diseases.

**Abbreviations**
BMP: bone morphogenetic protein
CVD: cardiovascular disease
EC: endothelial cell
EP: endothelial progenitor
EPC: endothelial progenitor cell
GREM1: Gremlin1
hESC: human embryonic stem cell
hiPSC: human induced pluripotent stem cell
hMSC: human mesenchymal stem cell
hPSC: human pluripotent stem cell
hPSC‐EP: human pluripotent stem cell induced endothelial progenitor
hUiPSC: human urinary induced pluripotent stem cell
hUiPSC‐EP: hUiPSCs‐induced endothelial progenitor

## 1 INTRODUCTION

Accumulating evidence suggest that bone marrow‐derived circulating endothelial progenitor cells (EPCs) contribute to vascular repair. Recently, EPC transplantation has become an experimental therapy for ischaemic disease.^1^ Several studies showed that EPC transplantation provides benefit for myocardial infarction^2^ and limb ischaemia.^3^, ^4^ However, the amount of endogenous EPCs is limited. In bone marrow or peripheral blood, the proportion of EPCs is only 0.01%. This number was even lower in cardiovascular patients.^5^

Human pluripotent stem cells (hPSCs) are ideal candidates for cell‐based regenerative repair, for they can self‐renew indefinitely and potentially differentiate into any cell type.^5^, ^6^ HPSC‐induced endothelial progenitors (hPSC‐EPs) may provide the means for vascularization of tissue‐engineered constructs and can serve as models to study vascular development and disease.^7^ Lian et al have reported a rapid and efficient method for the production of hPSC‐EPs and identifies Wnt/β‐catenin signalling as a primary regulator for generating vascular cells from hPSCs.^8^ This is a simple and efficient method for the conversion of hPSCs to CD34 + CD31+ endothelial progenitors. Appropriate temporal activation of regulators of Wnt signalling was sufficient to drive multiple hPSC to differentiate to CD34 + CD31+ endothelial progenitors. In another article, Bao et al also used chemically defined albumin‐free differentiation to induce human pluripotent stem cells to endothelial progenitor cells.^9^ Human urinary induced pluripotent stem cells (hUiPSCs), which are induced from exfoliated renal epithelial cells present in urine, provided a non‐invasive source for generating hPSCs. hUiPSCs also showed excellent differentiation potential and thus represent a good choice for producing pluripotent cells from normal individuals or patients with genetic diseases.^10^ In this study, established hUiPSCs were used to acquire hUiPSC‐induced endothelial progenitors (hUiPSC‐EPs) from non‐invasive source by Lian's method. Furthermore, we tried to study on the critical factors during the differentiation, improve the efficiency and clarify the mechanisms.

Gremlin1 (GREM1) is a pro‐angiogenic protein belonging to the cystine‐knot superfamily that includes transforming growth factor‐β proteins (TGF‐β) and the angiogenic vascular endothelial growth factors (VEGFs).^11^ GREM1 antagonizes bone morphogenetic proteins (BMPs) 2, 4 and 7, thereby preventing these ligands from interacting with their receptors.^12^

GREM1 could bind to vascular endothelial growth factor receptor 2 (VEGFR2) and promote angiogenesis.^10^ Our previous study showed that overexpression of GREM1 in human mesenchymal stem cells (hMSCs) has greater therapeutic effects against ischaemia compared with wild‐type hMSCs by enhancing the survival of hMSCs and endothelial cells (ECs).^13^ Other studies reported that GREM1 accelerates DMSO‐induced cardiomyogenesis through inhibition of the BMP‐signalling pathway.^14^ However, the effect of GREM1 in the differentiation of endothelial progenitors is currently unknown.

In this study, we tried to clarify the effects of GREM1 during hUiPSC‐EP differentiation and expansion. We acquired hUiPSC‐EPs in three specific stages, Stage 1 (Day 0‐2, mesoderm induction), Stage 2 (Day 2‐5, endothelial progenitors' induction) and Stage 3 (Day 5‐8, endothelial progenitors' expansion). To determine the critical role of GREM1 during hUiPSC‐EP differentiation and expansion, GREM1 gene was down‐regulated by RNA interfering or GREM1 recombinant protein was added in the three stages, respectively. Our study may increase the differentiation efficiency and function of hUiPSC‐EPs and provide a novel strategy to enhance stem cell‐based therapy for ischaemic diseases.

## 2 MATERIALS AND METHODS

### 2.1 Cell culture

Four pluripotent cell lines were used in this study. Two human urinary induced pluripotent stem cell (hUiPSC) lines (U1‐hUiPSC, U5‐hUiPSC) were kindly presented by Dr Guangjin Pan's group, who established these cell lines previously.^15^ Two human embryonic stem cell (hESC) lines (H1‐hESC, H9‐hESC) were established in our laboratory as described previously.^16^ Cells were maintained in mTeSR1 medium (STEMCELL Technologies) on matrigel‐coated plates (BD Biosciences).

### 2.2 Endothelial progenitors' differentiation

An established protocol was used for endothelial progenitors' differentiation of hUiPSC/hESCs without using VEGF.^8^, ^9^ Briefly, hUiPSC/hESCs were cultured on Matrigel‐coated six‐well plates in mTeSR1 medium to 80%‐90% confluence. The cells were dissociated into single cells with Accutase (Life Technologies). On Day 0, cells were treated with 6 μM CHIR99021 (Selleckchem) in DMEM/F12 medium with 100 μg/ml ascorbic acid (A8960, Sigma). On Day 2 of differentiation, CHIR99021‐containing medium was aspirated and DMEM/F12 medium with ascorbic acid was added and changed daily. On Day 5, hUiPSC/hESCs were successfully induced into endothelial progenitors. Cells were kept on expanding until Day 8.

### 2.3 GREM1 silencing with small interfering RNA (siRNA)

HUiPSCs or hESCs were transfected with double‐stranded GREM1 siRNA (siGREM1) or negative control siRNA (siCtrl) using Lipo2000 Transfection Reagent (Thermo Fisher Scientific). Target sequences were as follow: 5′‐CATCGATTTGGATTAAGCC‐3′ for GREM1. Double‐stranded siRNAs were synthesized by RiboBio (Guangdong). The details were shown as follows: siRNA‐GREM1 (sense) 5' CAUCGAUUUGGAUUAAGCC dTdT 3‘ (anti‐sense) 3‘ dTdT GUAGCUAAACCUAAUUCGG 5’. siGREM1(20μM) or siCtrl was added at day0, day2 or day5, respectively, for the knock‐down of GREM1 in different stage. Cells of specific stage were harvested to assess silencing efficacy of the stage.

### 2.4 Immunofluorescence (IF)

Cells were fixed in 4% (v/v) paraformaldehyde for 20 min and then permeabilized by incubation for 30 min at room temperature in PBS containing 0.1% (v/v) Triton X‐100, goat serum and 1% (w/v) bovine serum albumin (BSA; Sigma). Next, the cells were incubated overnight at 4°C with primary antibodies against CD31 (1:200; CST); CD34 (1:200; Abcam); CD144 (1:50; SantaCruz); VEGFR2 (1:200; CST); pVEGFR2 (Tyr1054, Tyr1059) polyclonal antibody (1:100, Thermo Fisher); pVEGFR2(pTyr1175) (1:100; CST); and vW Factor antibody (1:50; Santa cruz). Alexa Fluor 488‐ or Alexa Fluor 594‐conjugated secondary antibodies (1:1000, anti‐rabbit or antimouse; Thermo Fisher Scientific) were added to the samples and incubated at room temperature for 1 hours in the dark. The nuclei were counterstained with DAPI (1:1000; Sigma), and the cells were observed by Zeiss IOL Master 700 microscope (Carl Zeiss Meditec AG).

### 2.5 Flow cytometry (FACS)

Cells were dissociated into single cells with Accutase for 10 minutes at day 2, 5 or 8 in different experiments. The cell suspensions were incubated with fluorescent conjugated antibodies (Table S1) for 60 minutes at room temperature. Following incubation, the cells were re‐suspended in PBS containing 2% FBS. For the apoptosis assay, cells were treated with 1 mM H_2_O_2_ for 3 hours PI/Annexin V were detected. A cell sorting analysis was performed with a FACS Caliber Flow Cytometer (BD Bioscience), and the data were analysed using the Cell Quest‐Pro software (BD Bioscience).

### 2.6 Tube formation assay

To assess the formation of capillary structures, 1 × 10^5^ hUiPSC‐EPs or hESC‐EPs were suspended in 0.4 mL EGM‐2 medium (Lonza) were plated into one well of 24‐well tissue culture plate pre‐coated with Matrigel (BD Bioscience). Tube formation was observed by light microscopy after 24 hour of incubation. Tube length was calculated and quantified by Image J software (the National Institute of Health).

### 2.7 Ac‐LDL uptake assay

To assess the ability of cells to incorporate acetylated low‐density lipoprotein (Ac‐LDL), cells were incubated with 10 μg/mL of Ac‐LDL labelled with 1,1′‐dioctadecyl‐3,3,3′,3′‐tetramethylindocarbocyanine perchlorate (DiI, Molecular Probes) in EGM‐2 medium containing 10% (v/v) HyClone FBS (GE healthcare) for 4 hours at 37°C. Cells were washed 3 times with PBS, and then, the uptake of Ac‐LDL was confirmed using a Zeiss IOL Master 700 microscope (Carl Zeiss Meditec AG).

### 2.8 Quantitative reverse‐transcription PCR (qRT‐PCR)

Total RNA was extracted from cells using TRIzol Reagent (Thermo Fisher Scientific). Samples (1 μg) of total RNA were reverse‐transcribed using a First Strand complementary DNA synthesis kit for RT‐PCR (Roche). qRT‐PCR was performed with SYBR PCR Master Mix (Toyobo) according to the manufacturer's instructions. qRT‐PCR was conducted in duplicate for each sample, and three independent experiments were performed. Signals were detected using a Light Cycler 480 detection system (Roche). The primer sequences are listed (Table S2).

### 2.9 Western blot analysis

Cell lysates with equal total protein amounts were separated by SDS‐PAGE gel. Proteins were transferred electrophoretically to polyvinylidene difluoride (PVDF) membranes (Bio‐Rad). The membranes were blocked in 5% milk in PBS‐T (0.1% Tween 20) at room temperature for 1 hours The membranes were probed with primary antibody overnight (Table S3). The primary antibody was then identified by a horseradish peroxidase (HRP)‐conjugated secondary antimouse or anti‐rabbit antibody (1:1000; CST). Finally, the membranes were developed using an enhanced chemiluminescence advance detection kit (GE Healthcare) and exposed to x‐ray films. The band density was analysed using Image J software (the National Institute of Health).

### 2.10 Statistical analysis

All data are presented as the mean ± SEM obtained from at least three independent experiments. Comparisons between groups were performed with a one‐way analysis of variance (ANOVA). *P* < .05 was considered statistically significant. All statistical analyses were performed with the aid of SPSS Version 17.0 (SPSS Inc).

## 3 RESULTS

### 3.1 Stage‐specific expression of GREM1 during the differentiation and maintenance of hUiPSCs into endothelial progenitors

**Figure 1:**
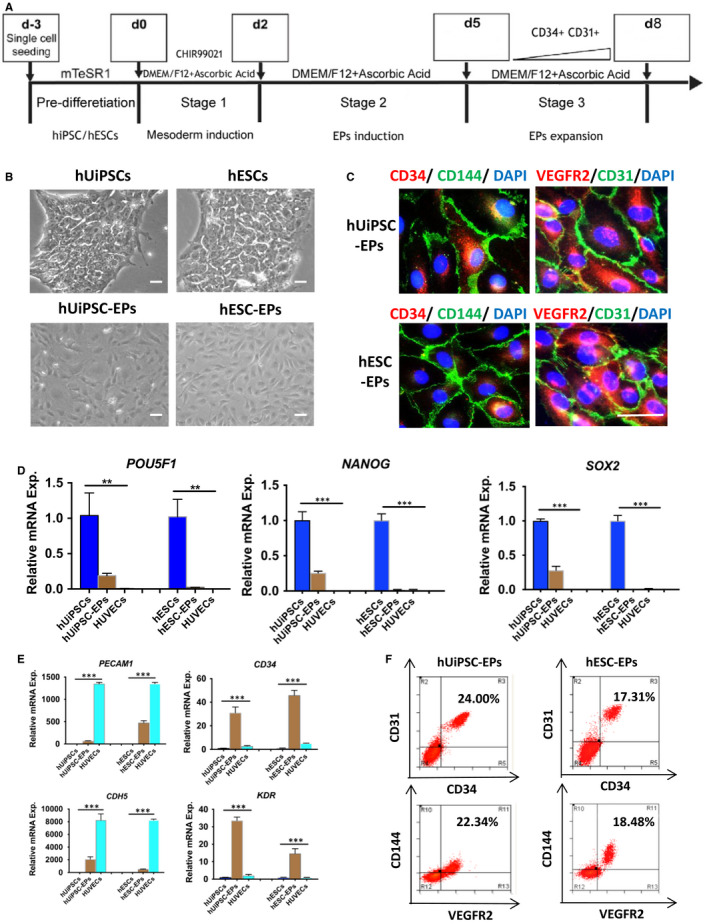
Characterization of hUiPSC‐EPs or hESC‐EPs. A, Schematic of the protocol for defined, growth factor‐free differentiation of hUiPSC/hESCs to endothelial progenitors in a single differentiation medium. B, Morphological characterization of hUiPSCs/hESCs and hUiPSC‐EPs/hESC‐EPs. C, Endothelial progenitors were verified by staining with CD34, CD31, VEGFR2 and CD144 for hUiPSC‐EPs/hESC‐EPs. D, Gene expression of hPSC markers, POUSF1, NANOG and SOX2 was detected by qRT‐PCT. E, Gene expression of endothelial progenitors markers, PECAM1, CD34, CDH5 and KDR was also tested. F, Co‐expression of CD34/VEGFR2 or CD34/CD144 for endothelial progenitors was detected by FACS. The data represent mean ± SEM of three independent experiments. *P < .05. Scale bar: 50 μm

To explore a non‐invasive source of endothelial progenitors, we induced hUiPSCs (U1‐hUiPSCs, U5‐hUiPSCs) into endothelial progenitors. H1‐hESCs and H9‐hESCs were also induced as control. Firstly, hUiPSC/hESCs were directed to mesoderm by CHIR99021 treatment for 2 days (Day 0‐2; Stage 1). Then, the cells were induced into endothelial progenitors by DMEM/F12 medium supplemented with ascorbic acid for 3 days (Day 2‐4; Stage 2). Finally, the cells were expanded (Day 5‐8; Stage 3) (Figure 1A). After respective treatment, hUiPSC/hESCs changed into endothelial progenitors (hUiPSC‐EPs or hESC‐EPs) (Figure 1B). At Day 5 of the differentiation, IF was applied to detect the expression of EPC surface markers, CD34, CD31, VEGFR2 and CD144 (Figure 1C). QRT‐PCR results showed hPSC markers OCT4, Nanog and SOX2 decreased after differentiation (Figure 1D). On the contrary, EPC markers, CD34, CD31, CD144 and VEGFR2 increased (Figure 1E). The expression of hPSC and EPC markers on each day showed similar results (Figure S1). FACS analysis confirmed the success of EP differentiation, as indicated by CD34/CD31 and VEGFR2/CD144 double positive rates (Figure 1F). We found that the protein expression of endothelial progenitors, especially CD34 and VEGFR2, was significantly increased after differentiation (Figure S2).

**Figure 2:**
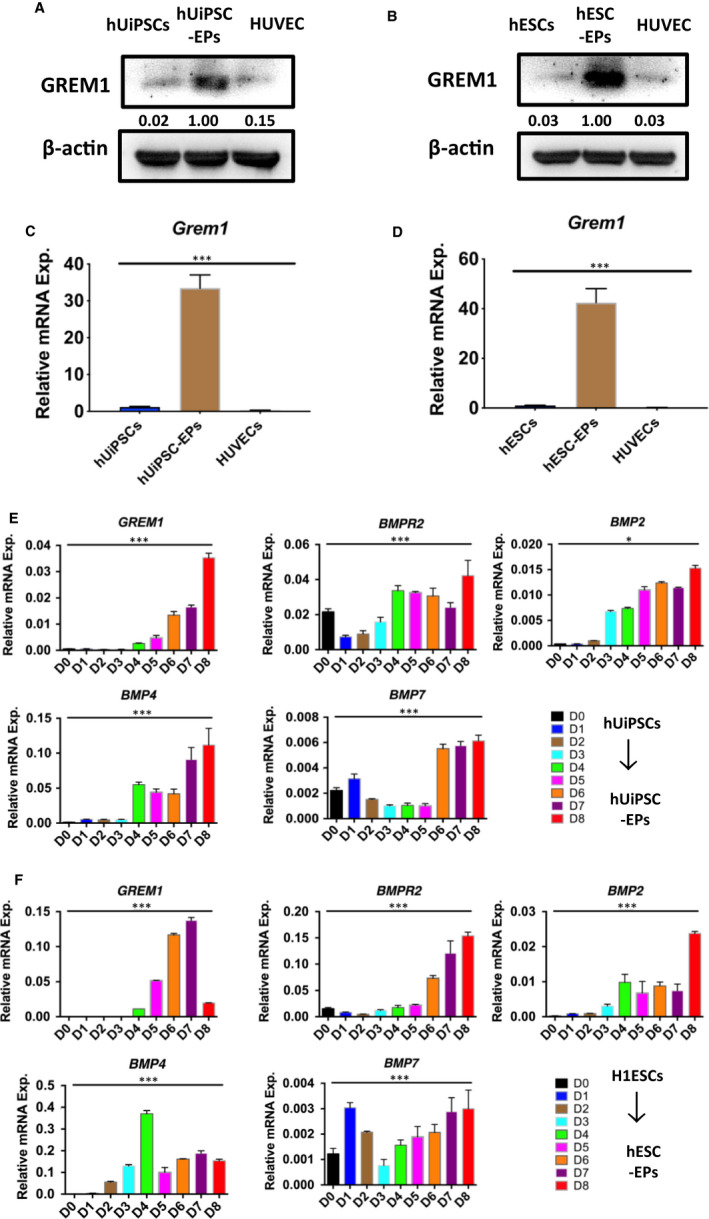
GREM1 expression during hUiPSC/hESCs‐EPs cell differentiation. A, GREM1 protein expression of hUiPSC‐EPs was determined by WB. B, GREM1 protein expression of hESC‐EPs was determined by WB. C, GREM1 mRNA expression of hUiPSC‐EPs was detected by qPCR. D, GREM1 mRNA expression of hESC‐EPs was detected by qPCR. E, RNA samples were collected at successive differentiation days for hUiPSC‐EPs, and expression of GREM1 and related genes were tested by qPCR. F, GREM1 and related genes were tested in hESC‐EPs by qPCR. The data represent mean ± SEM of three independent experiments. * P < .05

To clarify the mechanism of differentiation, GREM1 expression during the process was detected by WB and qRT‐PCR. WB results revealed there was few GREM1 protein expression in hUiPSCs or hESCs, while high GREM1 expression was detected in endothelial progenitors (Figure 2A &B). QRT‐PCR results showed GREM1 obviously increased after differentiation from hUiPSCs or hESCs into endothelial progenitors (Figure 2C &D). Furthermore, we detected daily mRNA expression of GREM1, BMPR2, BMP2, BMP4 and BMP7 during the differentiation. GREM1 mRNA expression was relatively low during Stage 1, but it steadily increased in Stage 2 and peaked at Stage 3. GREM1 expression followed a trend of reaching a peak and then decreasing slowly; this trend was similar in hUiPSCs (peak at Day 8) and hESCs (peak at Day 7) (Figure 2E,F and Figure S3).

GREM1 has been reported to be binding and inhibition of BMPs.^17^ However, the precise interactions between GREM1 and BMPs during hUiPSC‐EP differentiation and expansion have not been accurately defined. Hereby, BMPR2, BMP2, BMP4 and BMP7 were tested. The expression of BMP2 and BMP7 was negligible as compared to BMP4 during the differentiation. In mesoderm induction stage (Stage 1), BMP4 kept moderate expression. It reached the first peak during endothelial progenitors' induction stage (Stage 2) and then decreased. BMP4 expression reached to the second peak in endothelial progenitors' expansion stage (Stage 3). The expression of BMPR2 was consist to that of BMP4 (Figure 2E,F).

### 3.2 Knock‐down of GREM1 during Stage 1 promoted the differentiation and expansion of hUiPSCs into endothelial progenitors

**Figure 3:**
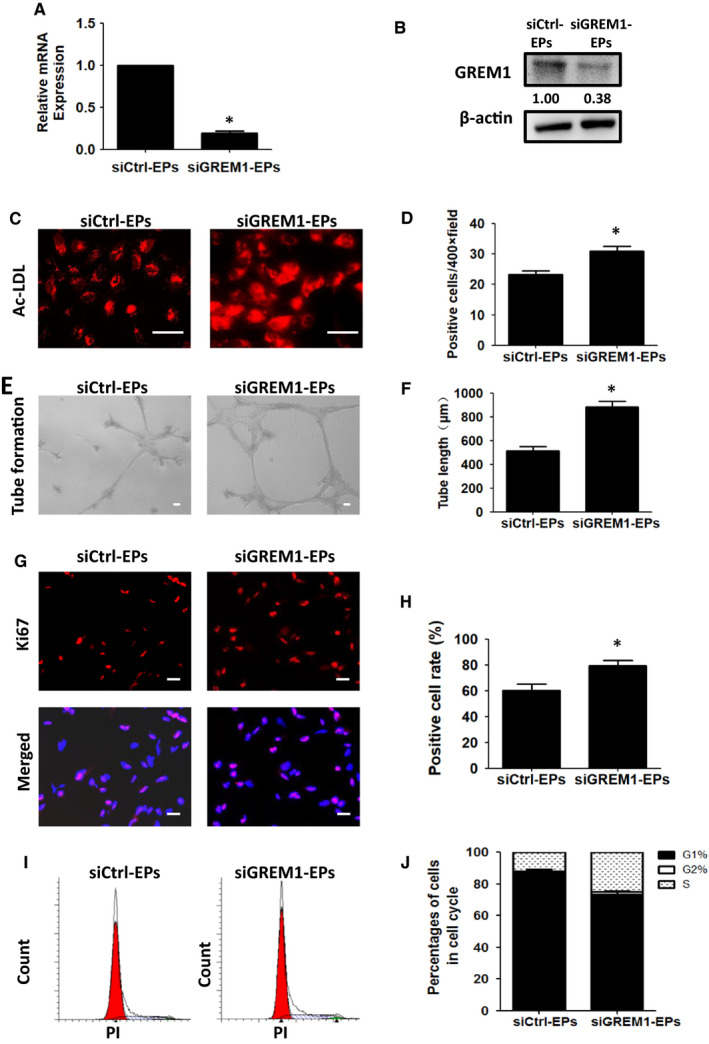
Knock‐down of GREM1 during Stage 1 promoted the differentiation and expansion of EPs. A, GREM1 mRNA expression was detected by qPCR in siCtrl‐EPs and siGREM1‐EPs. B, GREM1 protein was determined by WB. C, Ac‐LDL uptake in siGREM1‐EPs and siCtrl‐EPs was detected. D, Quantified data were analysed. E, Tube formation in siGREM1‐EPs or siCtrl‐EPs was detected. F, Quantified data were analysed. G, Ki67 expression was tested by immunofluorescence. H, Quantified data were analysed. I, Cell cycle was detected by FACS. J, Quantified data were analysed. The data represent mean ± SEM of three independent experiments. *P < .05. Scale bar: 50 μm

Although GREM1 mRNA expression was relatively low, it was knock‐down in Stage 1 to clarify the effects during mesoderm induction stage. At Day 2, the expression of GREM1 mRNA could be detected (Ct value was around 27), although the protein level of GREM1 protein was too low to be detected. Therefore, we proceeded to change the experimental design. siGREM1 was still added at Day 0 and removed 8 hours later. EP induction was kept on until Day 5. Cells were then harvested on Day 5. GREM1 mRNA (Ct value was around 23) and protein could be detected at this time‐point. The expression of GREM1 mRNA and protein was both significantly reduced in siGREM1‐EP group. Knock‐down of GREM1 siGREM1 indicated ~ 80% silencing efficacy as determined by qRT‐PCR (Figure 3A). The expression of GREM1 protein confirmed the result of mRNA (Figure 3B).

When GREM1 was silenced in Stage 1 (Day 0‐2), Ac‐LDL positive cells were increased from (23.33 ± 1.20) to (31.00 ± 1.53), *P* < .05 (Figure 3C,D). Tube formation of endothelial progenitors treated with siGREM (siGREM1‐EPs) increased to (883.30 ± 51.35) μm as compared to the endothelial progenitors treated with control siRNA (siCtrl‐EPs) (516.70 ± 33.21) μm, *P* < .05 (Figure 3E,F).

Simultaneously, siGREM1 treated cells indicated increased cell proliferation by IF and FACS. IF of Ki67 expression showed the positive cell rate in siGREM1‐EPs increased to (79.66 ± 3.79)% as compared to the siCtrl‐EPs (60.32 ± 4.98)%, *P* < .05 (Figure 3G,H). Cell cycle detected by FACS showed that cell ratio at G1 phase decreased from (86.40 ± 1.85)% to (79.40 ± 0.92)%, *P* < .05, while cells in S phase increased to (18.80 ± 0.73)% as compared to the siCtrl‐EPs (12.55 ± 1.82)%, *P* < .05 (Figure 3I,J).

### 3.3 Knock‐down of GREM1 during Stage 2 inhibited the differentiation of hUiPSCs into endothelial progenitors

**Figure 4:**
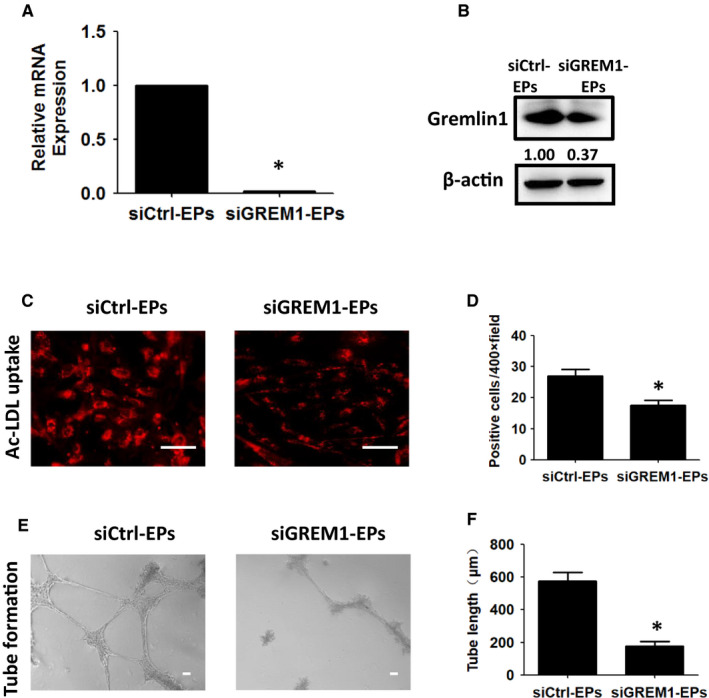
Knock‐down of GREM1 during Stage 2 inhibits the differentiation of EPs. A, GREM1 mRNA expression was detected by qPCR in siCtrl‐EPs and siGREM1‐EPs. B, GREM1 protein was determined by WB. C, Ac‐LDL uptake in siGREM1‐EPs or siCtrl‐EPs was detected. D, Quantified data were analysed. E, Tube formation in siGREM1‐EPs or siCtrl‐EPs was detected. F, Quantified data were analysed. The data represent mean ± SEM of three independent experiments. *P < .05. Scale bar: 50 μm

The roles of GREM1 during endothelial progenitors' induction stage, Stage 2, were tested by qRT‐PCR and WB. The efficiency of siGREM1 was confirmed by qRT‐PCR and WB (Figure 4A,B). The Ac‐LDL uptake positive cells were decreased from (27.00 ± 2.08) to (17.67 ± 1.45), *P* < .05 (Figure 4C,D). Tube formation decreased to (178.00 + 27.15) μm as compared to the siCtrl‐EPs from (575.80 ± 53.99) μm, *P* < .05 (Figure 4E,F).

**Figure 5:**
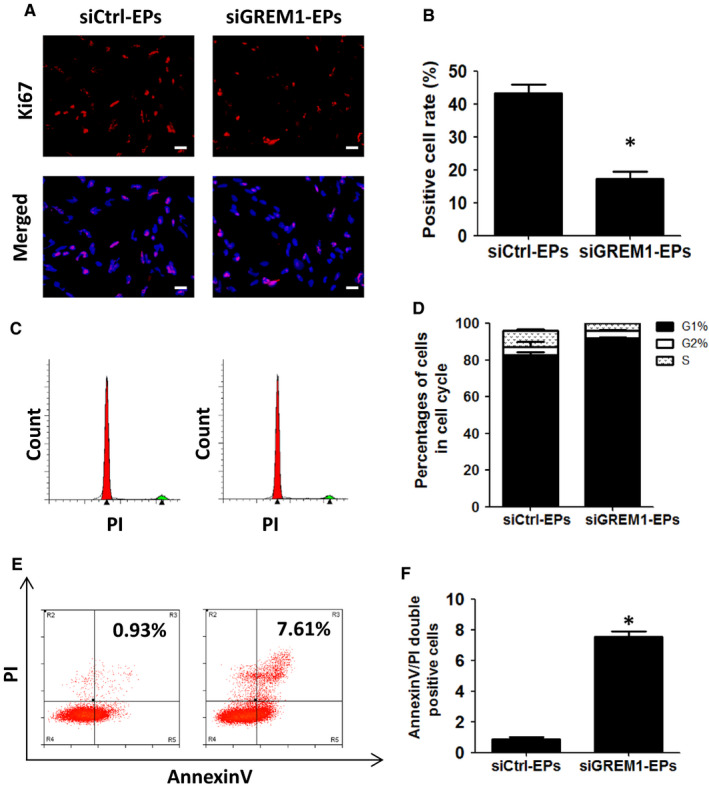
Knock‐down of GREM1 during Stage 2 inhibits proliferation, but promoted apoptosis. A, Ki67 expression was tested by immunofluorescence. B, Quantified data were analysed. C, Cell cycle was detected by FACS. D, Quantified data were analysed. E, Cell apoptosis was tested by PI/Annexin V. F, Quantified data were analysed. The data represent mean ± SEM of three independent experiments. *P < .05. Scale bar: 50 μm.

The proliferation and apoptosis of endothelial progenitors were also detected. Ki67 expression showed the positive cells were decreased from (43.43 ± 2.63)% to (17.33 ± 2.17)%, *P* < .05 (Figure 5A,B). Cell cycle detected by FACS showed that cell ratio at G1 phase increased from (82.71 ± 1.44) % to (91.82 ± 0.64)% and decreased from (9.07 ± 0.66) % to (4.16 ± 0.14) % at S phase (Figure 5C,D). PI/AnnexinV results showed that double positive rate increased from (0.89 ± 0.11)% to (7.58 ± 0.37)%, *P* < .05 (Figure 5E,F).

### 3.4 Knock‐down of GREM1 during Stage 3 inhibits the function and expansion of hUiPSCs into endothelial progenitors

**Figure 6:**
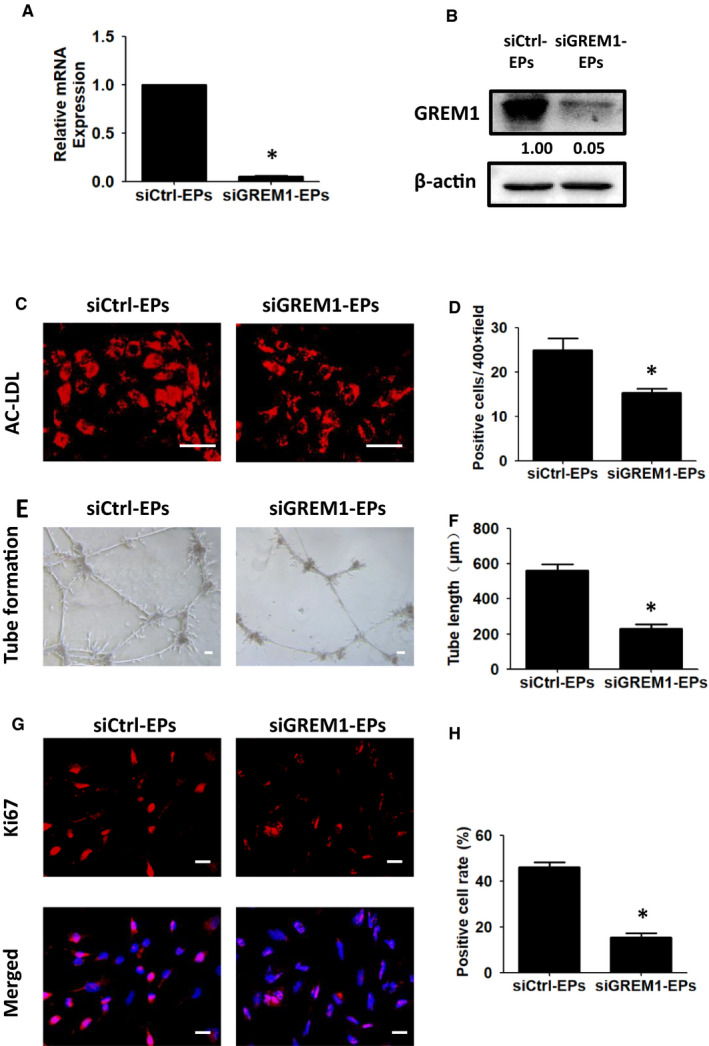
Knock‐down of GREM1 during Stage 3 inhibits EPs differentiation and proliferation. A, GREM1 mRNA expression was detected by qPCR in siCtrl‐EPs and siGREM1‐EPs. B, GREM1 protein was determined by WB. C, Ac‐LDL uptake in siGREM1‐EPs and siCtrl‐EPs was detected. D, Quantified data were analysed. E, Tube formation in siGREM1‐EPs or siCtrl‐EPs was detected. F, Quantified data were analysed. G, Ki67 expression was tested by immunofluorescence. H, Quantified data were analysed. The data represent mean ± SEM of three independent experiments. *P < .05. Scale bar: 50 μm

The effects of GREM1 were then detected by using siGREM1 during Stage 3. The interfering efficiency of siGREM1 was confirmed by qPCR and WB (Figure 6A,B). The Ac‐LDL uptake positive cells decreased from (25.00 ± 2.65) to (15.33 ± 0.88), *P* < .05 (Figure 6C,D). Tube formation decreased from (561.40 ± 36.31) μm to (230.00 + 23.85) μm, *P* < .05 (Figure 6E,F). The proliferation and apoptosis were also detected. Ki67 expression showed the positive cells decreased from (46.03 ± 2.26)% to (15.50 ± 1.83)%, *P* < .05 (Figure 6G,H).

### 3.5 Stage‐specific addition of recombinant protein GREM1 influenced the differentiation and expansion of hUiPSCs into endothelial progenitors

**Figure 7:**
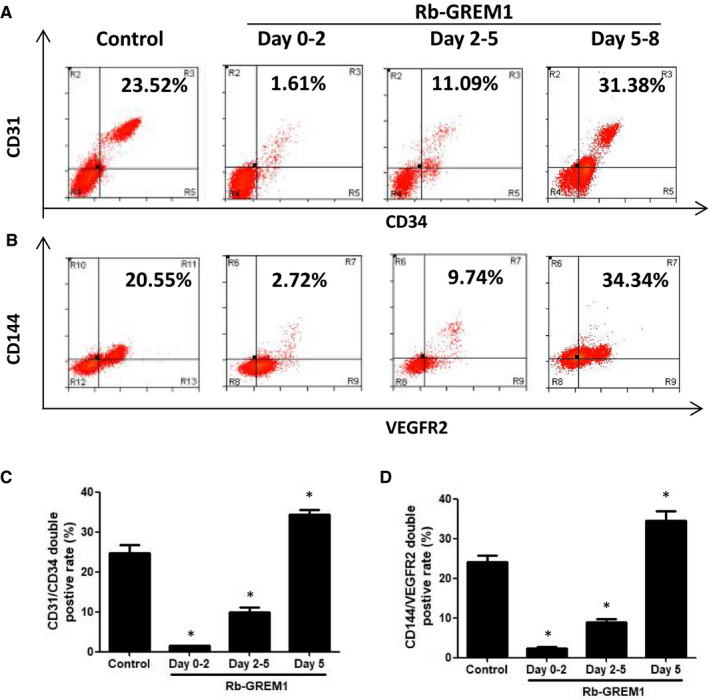
Stage‐specific addition of recombinant protein GREM1 influenced EPs differentiation and maintenances. A, Recombinant protein GREM1 was added during day 0‐2, 2‐5 or 5 of the differentiation, and CD34/CD31 was detected by FACS. B, VEGFR2/CD144 was tested by FACS. C, Quantified data of CD31/CD34 were shown. D, Quantified data of VEGFR2/CD144 were shown. The data represent mean ± SEM of three independent experiments. *P < .05

To improve the differentiation and expansion of endothelial progenitors, we used recombinant protein GREM1 during different stages, respectively. We determined that the addition of GREM1 during Stage 1 significantly decreased endothelial progenitors' generation (CD31/34 co‐expression, (2.24 ± 0.48)% vs. (24.17 ± 1.49)%, *P* < .05). When GREM1 was added during Stage 2, the differentiation efficiency decreased to (8.85 ± 0.90)%. Addition of GREM1 during Stage 3 increased the differentiation efficiency to (34.52 ± 2.37)% (Figure 7A,C). The results of VEGFR2/CD144 had similar change (Figure 7B,D).

### 3.6 Recombinant protein GREM1 during Stage 3 promoted endothelial progenitors' expansion and activated the downstream pathway

**Figure 8:**
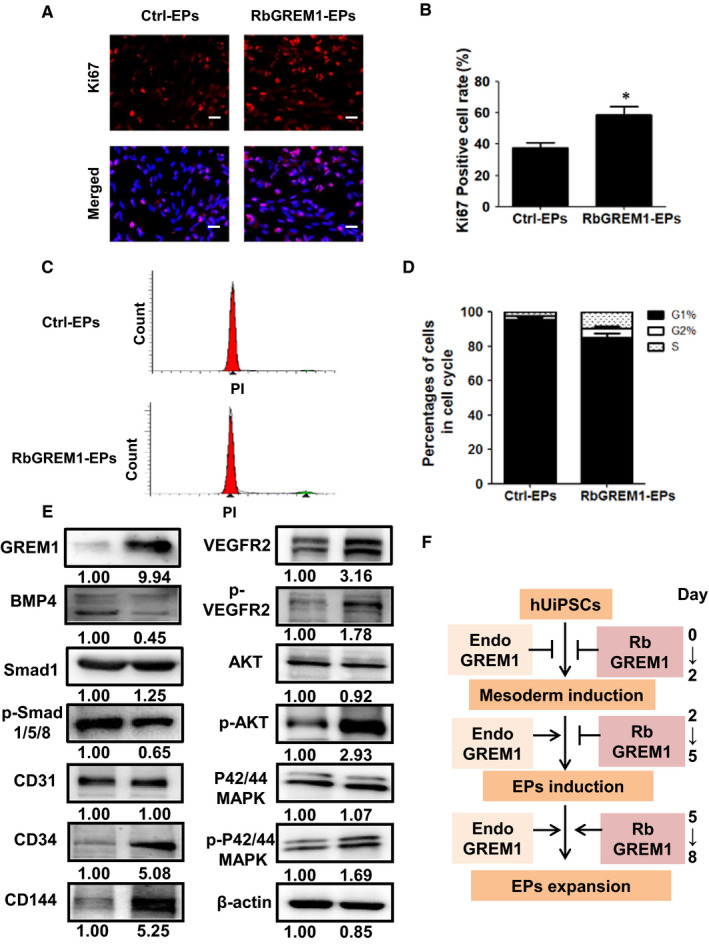
Recombinant protein GREM1 during Stage 3 promoted cell proliferation and activated downstream pathway. A, Ki67 expression was tested by immunofluorescence. B, Quantified data were analysed. C, Cell cycle was detected by FACS. D, Quantified data were analysed. E, GREM1 and related proteins were determined by WB. F, Schematic model of the effect of GREM1 during endothelial progenitors' differentiation and maintenance. Endo GREM1: endogenous GREM1. The data represent mean ± SEM of three independent experiments. *P < .05. Scale bar: 50 μm

As addition of GREM1 during Stage 3 increased the differentiation efficiency, we detected the role of GREM1 during this maintenance of endothelial progenitors’ stage. GREM1 was added during Stage 3. Ki67 expression showed the positive cells increased from (34.56 ± 1.55)% to (62.21 ± 1.94)%, *P* < .05 (Figure 8A,B). Cell cycle showed that cell ratio at G1 phase decreased from (95.73 ± 1.14)% to (85.08 ± 2.62)% and increased from (2.42 ± 0.91)% to (9.46 ± 1.50)% at S phase in Rb‐GREM1 group (Figure 8C,D). To clear if the proliferative cells were endothelial cells, CD144 staining was added. We found that GREM1 could increase the positive rates of Ki67 both in endothelial and non‐endothelial cells (Figure S4).

To determine the mechanism through which GREM1 regulates endothelial progenitors' maintenance, we evaluate the canonical signal transduction pathway VEGFR2/Akt and VEGFR2/p42/44MAPK. We found addition of recombinant protein GREM1 during Stage 3 increased the phosphorylation of VEGFR2, Akt and p42/44MAPK (Figure 8E), while BMP4 and its downstream phospharated‐smad1/5/8 were down‐regulated. To make sure the phosphorylation of VEGFR2, immunofluorescence was used. Recombinant protein GREM1 could induce phosphorylation of VEGFR2 in endothelial cells (Figure S5). Three different inhibitors, VEGFR2 inhibitor (SU1498, 10 μM), Akt inhibitor (ADJ6244, 20 μM) and p42/44MAPK inhibitor (PD98059, 30 μM), were then used to test whether the signal pathways mediated the proliferative effects of GREM1. The staining of Ki67 showed the inhibitors partly reversed the increase of Ki67 promoted by GREM1, which suggested the involvement of VEGFR2/Akt and VEGFR2/p42/44MAPK pathways in the proliferative effects of GREM1 (Figure S6).

Throughout all stages, GREM1 expression kept a concise balance during endothelial progenitors' differentiation and expansion. Schematic model of the effect of GREM1 during endothelial progenitors' differentiation and maintenance was shown (Figure 8F). A stage‐specific bipolar effect of GREM1 was demonstrated: decreasing hUiPSC‐EP differentiation in mesoderm induction stage (Stage 1), while increasing the hUiPSC‐EP differentiation in endothelial progenitors' induction stage (Stage 2) and expansion stage (Stage 3). We also found that the addition of recombinant protein GREM1 in endothelial progenitors' expansion stage (Stage 3) promoted the expansion of hUiPSC‐EPs, which was accomplished through VEGFR2/Akt and VEGFR2/p42/44MAPK pathway.

## 4 DISCUSSION

HiPSCs have been generated with varied efficiency from multiple tissues. Yet, acquiring donor cells is, in most instances, an invasive procedure that requires laborious isolation.^18^, ^19^ Zhou et al present a simple, reproducible, non‐invasive method for generating hiPSCs from renal tubular cells present in urine. The procedure eliminates many problems associated with other protocols, and the resulting hUiPSCs display an excellent ability to differentiate.^9^, ^20^ Dr Pan's laboratory also established hUiPSCs in Guangzhou, China. In this study, we carried out endothelial progenitors' differentiation on hUiPSCs and successfully acquired CD34 + CD31+ endothelial progenitors. This method provides a non‐invasive source of endothelial progenitors for the treatment of ischaemic diseases. However, the differentiation efficiency and the function of the hUiPSC‐EP were not satisfactory. Then, we tried to search the key gene during the process and improve the differentiation efficiency and the function.

Many studies have indicated that GREM1 is involved in cell differentiation and development, such as osteogenesis, myogenesis and limb formation.^21^, ^22^, ^23^ Our study proved that during the differentiation of hUiPSCs into endothelial progenitors, the expression of GREM1 continuously and steadily increased. To clarify the effect of GREM1 in different stages, GREM1 was silenced in the mesoderm induction stage (Stage 1). The results showed Ac‐LDL uptake and tube formation increased and promoted cell proliferation. These results suggested GREM1 inhibited endothelial progenitors' differentiation in the mesoderm induction stage. On the contrary, down‐regulation of GREM1 did benefit to the endothelial progenitors' differentiation.

It is reported that the BMP signalling is sufficient to activate the entire mesoderm progenitor gene signature. During development, BMP4 is a key regulatory factor, which also determines the differentiation direction of endothelial and hematopoietic cells, while inducing the differentiation of mesoderm in vitro.^24^ GREM1, the BMP antagonist, has been shown to inhibit BMP action in a range of different cell types and developmental stage‐specific contexts. In our study, knock‐down of GREM1 increased cell differentiation and proliferation. The effects may be completed by disinhibited BMP signal pathways, which accelerate mesoderm induction.

As the roles of GREM1 during different stages were diverse, we used recombinant protein GREM1, respectively, to improve hUiPSC‐EP differentiation efficiency and the function. During Stage 1, endogenous or recombinant protein GREM1 inhibited the differentiation. The reason might be the inhibition of GREM1 to BMP4 by competing BMPR2. During Stage 2, endogenous GREM1 promoted the differentiation. But the recombinant protein GREM1 inhibited the process. These suggested the concentration of GREM1 must be concise to regulate the balance between BMP4 and VEGFR2.

During Stage 3, both endogenous and recombinant protein GREM1 promoted endothelial progenitors' differentiation and maintenance. Recombinant protein GREM1 during Stage 3 promoted cell proliferation. Ki67 expression showed the positive cells increased. Using CD144 as endothelial marker, we found that GREM1 could increase the positive rates of Ki67 both in endothelial and non‐endothelial cells. This result suggested the non‐selective proliferation effect of GREM1, which could be studied extensively in the future.

Furthermore, we detected the signal pathway mechanism of GREM1 during the maintenance of endothelial progenitors. In our study, WB results showed recombinant protein GREM1 decreased BMP4 and its downstream signal factor p‐Smad1/5/8. Our previous study also proved GREM1 could bind to BMP4 and decrease the downstream signal pathway.^25^ Further study could be carried out to clarify the detail of interaction between GREM1 and BMP4. VEGFR2 was phosphorylated by recombinant protein GREM1. The PI3K/Akt and MAPK pathways promote endothelial cell proliferation, permeability, migration and tube formation in response to a variety of extracellular signals.^26^ We found addition of recombinant protein GREM1 during Stage 3 increased the phosphorylation of VEGFR2, Akt and p42/44MAPK, which were important in the proliferation. This is clear and interesting as a very recent report, highlighted that GREM1 did not induce phosphorylation in endothelial cells.^27^ To find out why the results are different, we used two different pVEGFR2 antibody; one was pVEGFR2 (Tyr1054, Tyr1059) polyclonal antibody, and the other was pVEGFR2(pTyr1175). Results indicated that the staining by pVEGFR2(pTyr1175) was negative, which was consistent with their results. However, the staining by pVEGFR2 (Tyr1054, Tyr1059) polyclonal antibody was positive after adding GREM1 recombinant protein. The results proved that GREM1 could induce phosphorylation of VEGFR2.

In conclusion, we provide a new non‐invasive source, exfoliated renal epithelial cells present in urine, for endothelial progenitors. A stage‐specific effect of GREM1 was demonstrated, and the addition of recombinant protein GREM1 in endothelial progenitors' expansion stage promoted the expansion of hUiPSC‐EPs, which was accomplished through VEGFR2/Akt and VEGFR2/p42/44MAPK pathway. These discoveries could lead to novel applications in the induction method of producing endothelial progenitors for the treatment of ischaemic disease.

## CONFLICT OF INTEREST

All authors declared no conflicts of interest.

## AUTHOR CONTRIBUTION

**Haixuan Chen:** Conceptualization (equal); Data curation (equal); Formal analysis (equal). **Zhen Zhang:** Conceptualization (equal); Data curation (equal); Formal analysis (equal). **Zhecun Wang:** Conceptualization (equal); Data curation (equal); Formal analysis (equal). **Quhuan Li:** Formal analysis (equal). **Hui Chen:** Formal analysis (equal). **Song Guo:** Data curation (equal); Methodology (equal). **Lin Bao:** Data curation (equal); Methodology (equal). **Zheng Wang:** Data curation (equal); Writing‐review & editing (equal). **Wang Min:** Project administration (equal); Supervision (equal). **Qiuling Xiang:** Conceptualization (equal); Data curation (equal); Funding acquisition (equal); Project administration (equal); Writing‐original draft (equal). QLX and WM designed the study and wrote the manuscript. HXC, ZZ, ZZW, ZW, SG and LB performed the experiments. QHL and HC collected, analysed and interpreted data. All authors read and approved the final manuscript.

## ETHICAL APPROVAL

All experimental research on animals followed internationally recognized guidelines, the Declaration of Helsinki, and all guidelines in China.

## Supporting information

Fig S1‐S6

Table S1‐S3

## Data Availability

The data sets used and/or analysed during the current study are available from the corresponding author on reasonable request.
