# Supplementary material for: Stage‐specific regulation of Gremlin1 on the differentiation and expansion of human urinary induced pluripotent stem cells into endothelial progenitors
Source: J Cell Mol Med. 2020 May 28;24(14):8018–30. doi: 10.1111/jcmm.15433 (PMC7348142; doi:10.1111/jcmm.15433)
Supplement: Supplementary file 1 — Fig S1‐S6 [file JCMM-24-8018-s001.pdf]

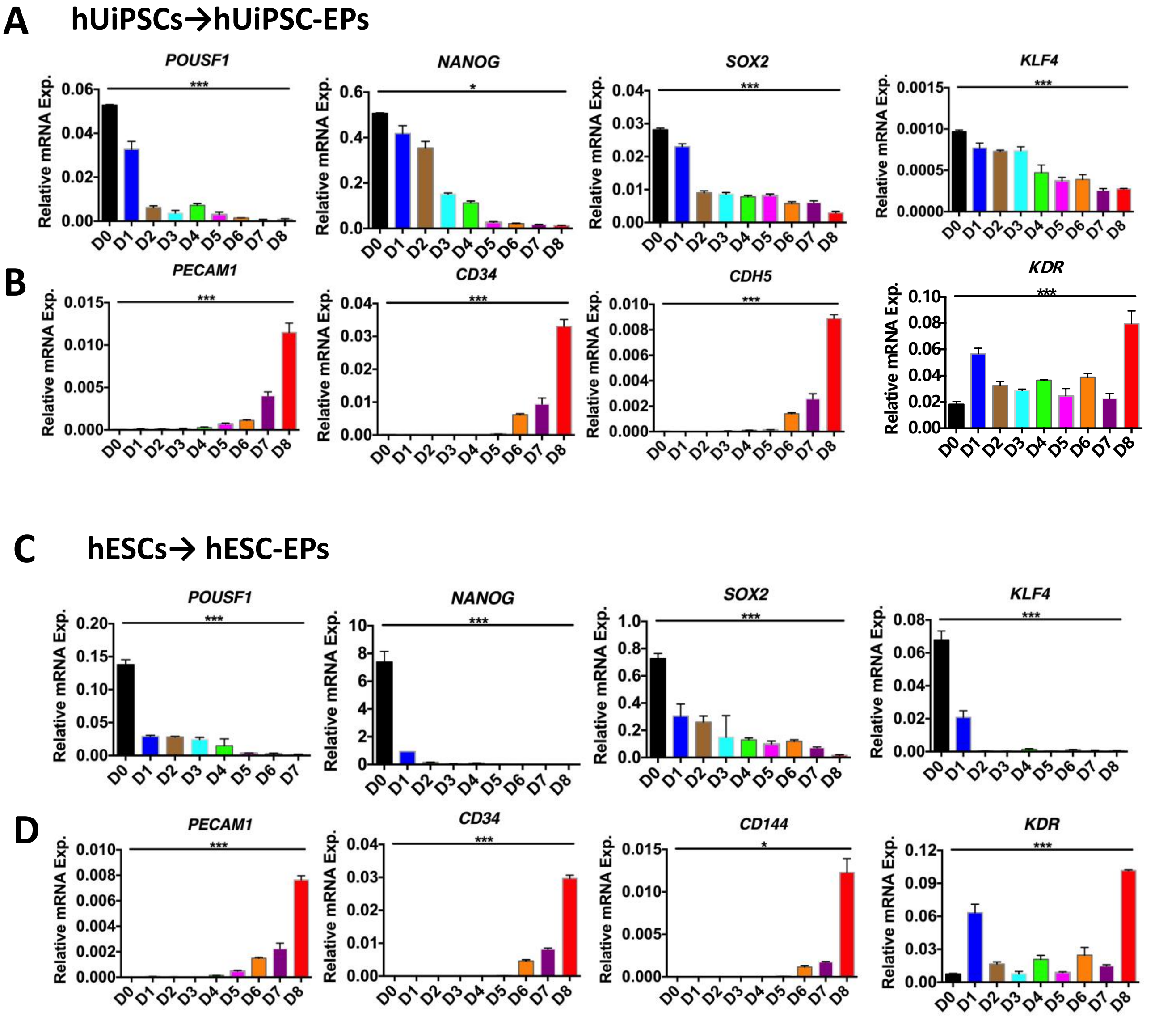

Fig. S1. HPSC-related genes and endothlial progenitor-related genes expression during hUiPSC-EPs or hESC-EPs differentiation.

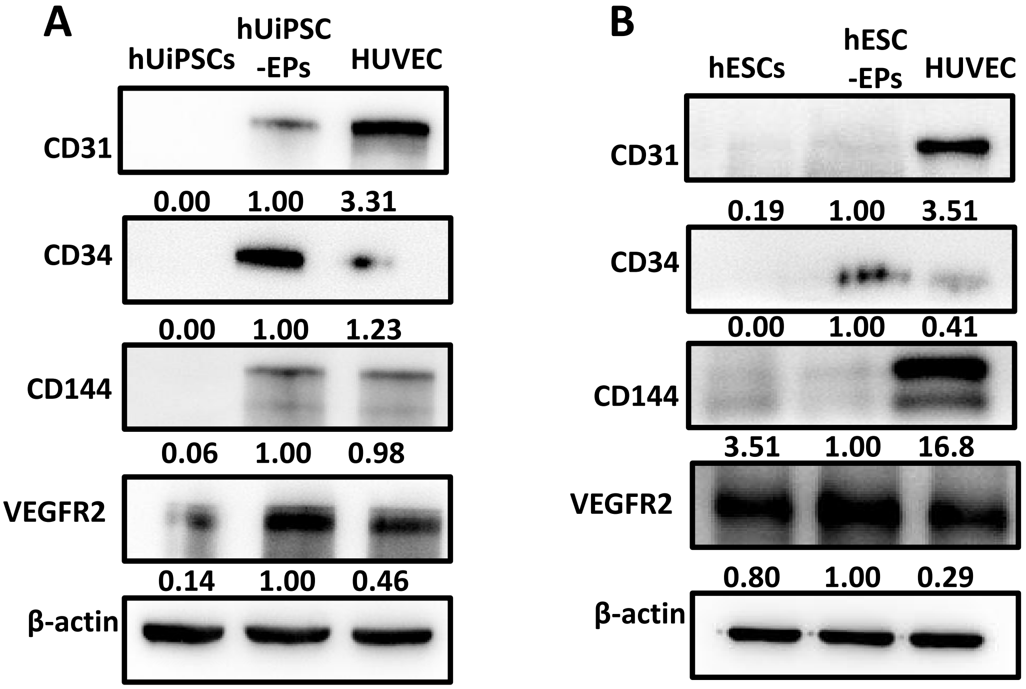

Fig. S2. Endothlial progenitor-related protein expression during hUiPSC-EPs or hESC-EPs differentiation.

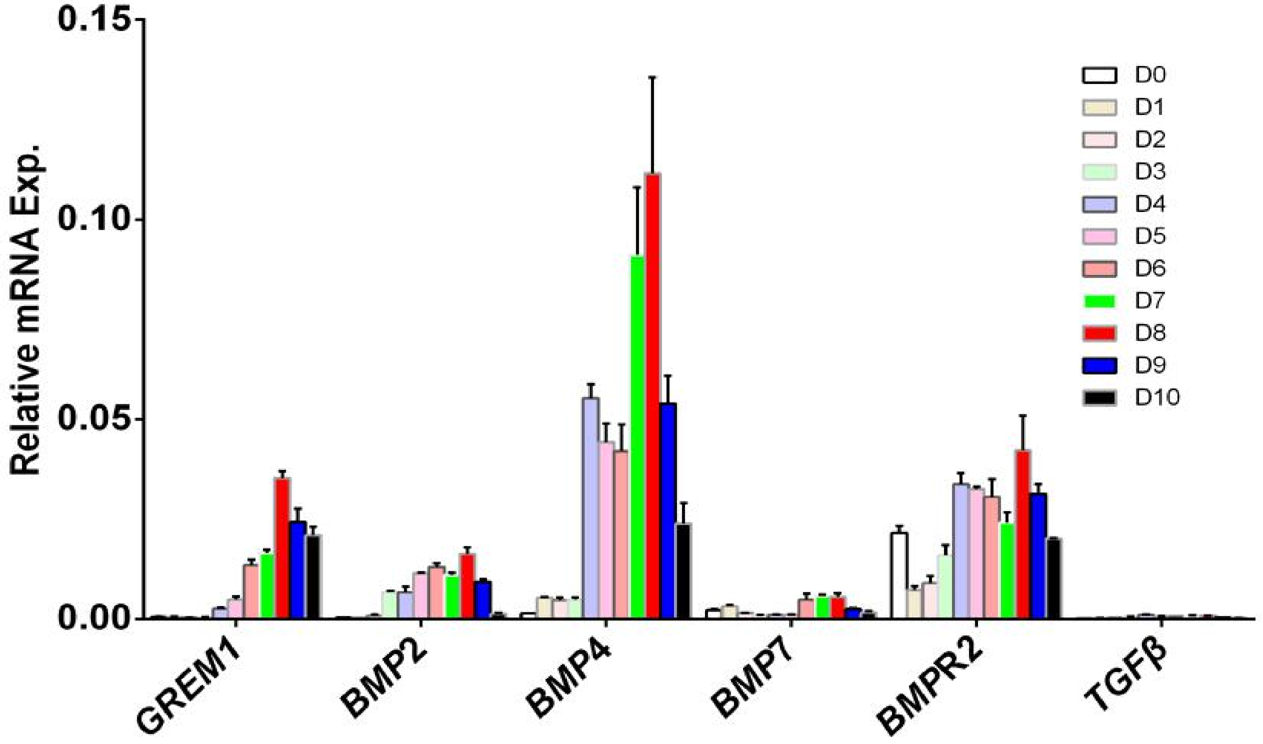

Fig. S3. GREM1 and related genes expression during hUiPSC-EPs differentiation.

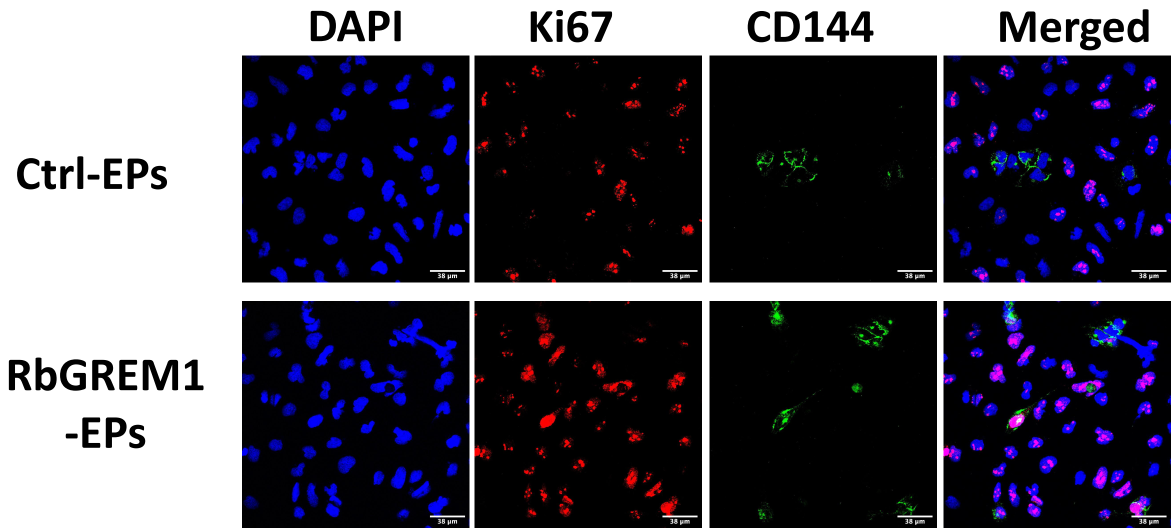

Fig. S4. Recombinant protein GREM1 during Stage 3 could promte proliferation in both endothelial and non-endothelial cells.

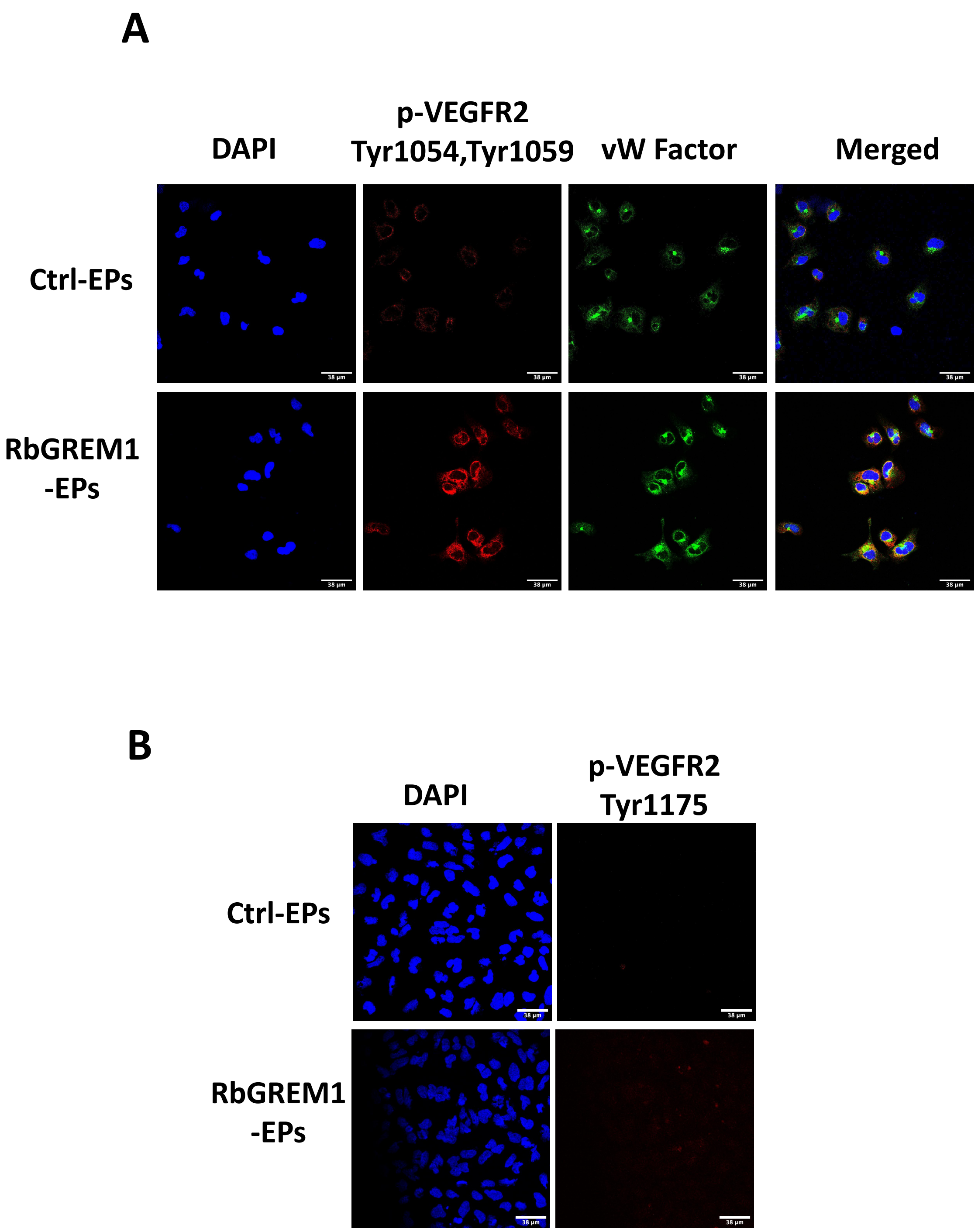

Fig. S5. Recombinant protein GREM1 during Stage 3 could induce phosphorylation of VEGFR2 in endothelial cells.

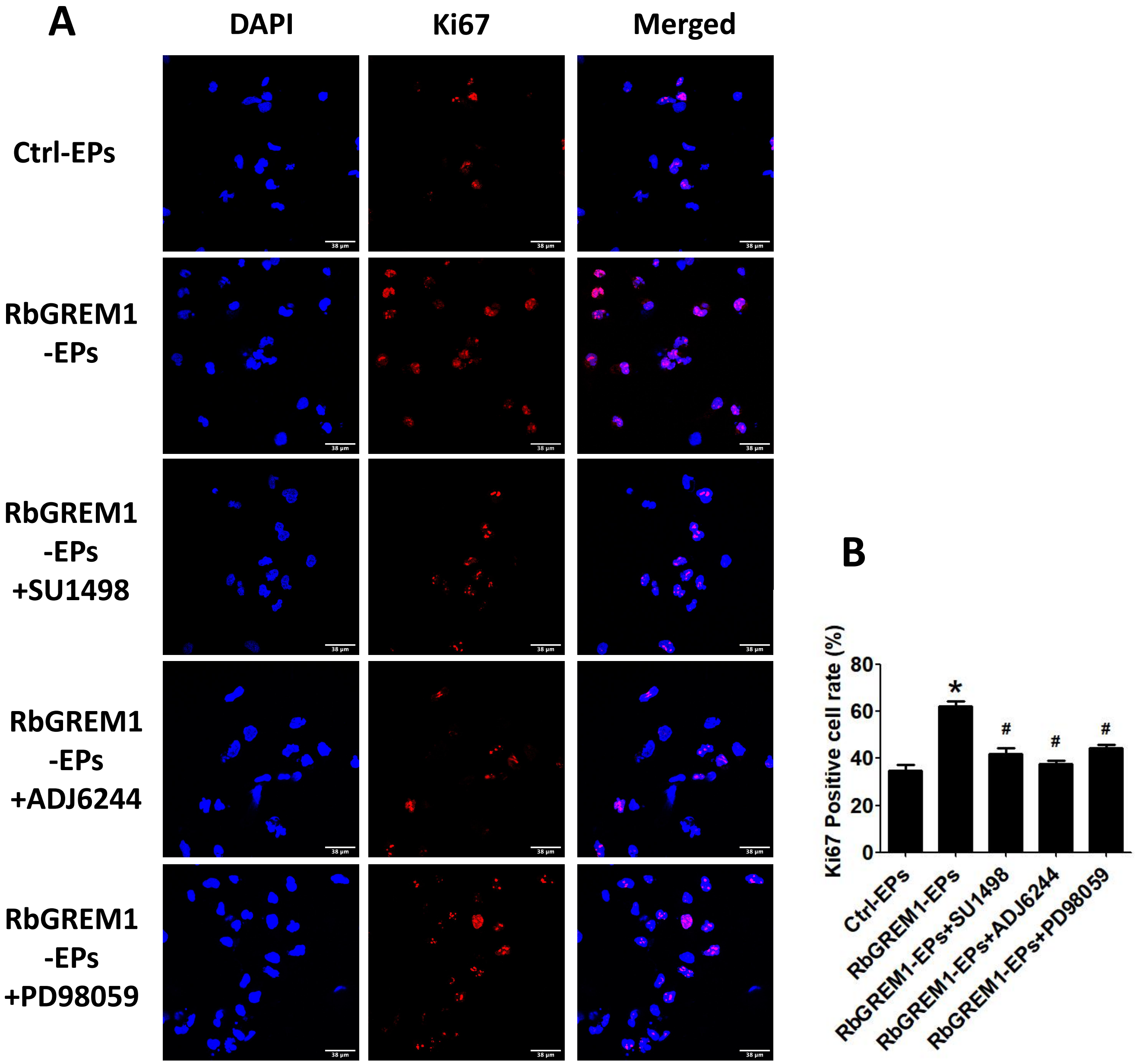

Fig. S6. The VEGFR2/Akt and VEGFR2/p42/44MAPK signal pathways mediated the effect of recombinant protein GREM1 during Stage 3.
