# Supplementary material for: Stage‐specific regulation of Gremlin1 on the differentiation and expansion of human urinary induced pluripotent stem cells into endothelial progenitors
Source: J Cell Mol Med. 2020 May 28;24(14):8018–30. doi: 10.1111/jcmm.15433 (PMC7348142; doi:10.1111/jcmm.15433)
Supplement: Supplementary file 2 — Table S1‐S3 [file JCMM-24-8018-s002.pdf]

Supplementary Table 1. Antibodies used in FACS

Antibodies	Host	Dilution	Cat. No.	Supplier
CD31	mouse	1:20	#1934035	Invitrogen
CD34	mouse	1:20	#343604	Biolegend
CD144	mouse	1:20	#348508	Biolegend
VEGFR2	mouse	1:5	#580494	BD Pharmingen ™

Supplementary Table 2. Primers for qPCR

Gene	primers	
GREM1	Forward	TTA AGCAGACCATCC ACGA
	Reverse	TGTAGTTCAGGGCAGTTGAGT
PECAM1	Forward	CCAAGGTGGGATCGTGAGG
	Reverse	TCGGAAGGATAAAACGCGGTC
CD34	Forward	TTTGCTTGCTGAGTTTGCTG
	Reverse	ATTTGAAAATGTTCCCTGGGT
CDH5	Forward	CATCTTCCCAGGAGGAACAG
	Reverse	AGAGCTCCACTCACGCTCAG
KDR	Forward	CCTGTATGGAGGAGGAGGAA
	Reverse	CGGCTCTTTCGCTTACTGTT
BMP2	Forward	CACTGTGCGCAGCTTCC
	Reverse	CCTCCGTGGGGATAGAACTT
BMP4	Forward	GCATTCGGTTACCAGGAATC
	Reverse	TGAGCCTTTCCAGCAAGTTT
BMP7	Forward	TCGGCACCCATGTTCATGC
	Reverse	GAGGAAATGGCTATCTTGCAGG
BMPR2	Forward	CGGCTGCTTCGCAGAATCA
	Reverse	TCTTGGGGATCTCCAATGTGAG
POU5F1	Forward	CTGGGTTGATCCTCGGACCT
	Reverse	CCATCGGAGTTGCTCTCCA
NANOG	Forward	TTTGTGGGCCTGAAGAAAACT
	Reverse	AGGGCTGTCCTGAATAAGCAG
SOX2	Forward	GCCGAGTGGAAACTTTTGTCG
	Reverse	GGCAGCGTGTACTTATCCTTCT
KLF4	Forward	CCCACATGAAGCGACTTCCC
	Reverse	CAGGTCCAGGAGATCGTTGAA
GAPDH	Forward	GGAGCGAGATCCCTCCAAAAT
	Reverse	GGCTGTTGTCATACTTCTCATGG

Supplementary Table 3. Primary antibodies

Primary antibodies	Host	Dilution	Cat. No.	Supplier
GREMLIN1	rabbit	1:1000	#4383	Cell Signaling Technology
CD31	mouse	1:1000	#3528	Cell Signaling Technology
CD34	rabbit	1:1000	#81289	Abcam
CD144	goat	1:500	#6458	Santa Cruz
VEGFR2	rabbit	1:1000	#9698	Cell Signaling Technology
BMP4	rabbit	1:1000	#124715	Abcam
p-VEGFR2（Tyr1212）	rabbit	1:1000	#2477	Cell Signaling Technology
AKT	rabbit	1:1000	#9272	Cell Signaling Technology
p-AKT	rabbit	1:1000	#2965	Cell Signaling Technology
P44/42 MAPK	rabbit	1:1000	#4695	Cell Signaling Technology
p- P44/42 MAPK	rabbit	1:1000	#4370	Cell Signaling Technology
SMAD2/3	rabbit	1:1000	#8685	Cell Signaling Technology
p-SMAD2/3	rabbit	1:1000	#8828	Cell Signaling Technology
TGFβ	rabbit	1:1000	#3709	Cell Signaling Technology
Ki67	rabbit	1:1000	#9129	Cell Signaling Technology
β-actin	rabbit	1:1000	#4970	Cell Signaling Technology
p-VEGFR2(Tyr1175)	rabbit	1:1000	#2478	Cell Signaling Technology
p-VEGFR2(Tyr1054, Tyr1059)	rabbit	1:1000	#44-1047-G	Thermofisher
vW Factor	mouse	1:1000	#sc53466	Santacruz
